# Transurethral cystoscopy in dogs with recurrent urinary tract infections: Retrospective study (2011‐2018)

**DOI:** 10.1111/jvim.15728

**Published:** 2020-02-26

**Authors:** Marie Llido, Catherine Vachon, Melanie Dickinson, Guy Beauchamp, Marilyn Dunn

**Affiliations:** ^1^ Department of Clinical Sciences, School of Veterinary Medicine University of Montreal Saint‐Hyacinthe Quebec Canada; ^2^ Department of Clinical Studies, Ontario Veterinary College University of Guelph Guelph Ontario Canada

**Keywords:** bacterial cystitis, cystoscopic findings, interventional urology, persistent infection, urinary incontinence

## Abstract

**Background:**

Urinary tract infections (UTIs) are common in female dogs and recurrent infections often require investigation by transurethral cystoscopy.

**Hypothesis/Objectives:**

Describe the findings of transurethral cystoscopy in dogs presented for recurrent urinary tract infections (RUTI).

**Animals:**

Fifty‐three client‐owned dogs with RUTI were included in the study.

**Methods:**

Retrospective study. Data collected from medical records included signalment, clinical findings, bladder wall culture, cystoscopic, and histopathologic findings. UTI was defined as: presence of compatible clinical signs and at least 2 out of 3 of the following criteria: (1) pyuria, (2) positive urine culture, (3) resolution of clinical signs with antibiotic treatment. Recurrence of UTI was defined as at least 2 episodes of UTI within 6 months or at least 3 or more in 1 year.

**Results:**

The mean age at presentation was 3.8 years with a majority of female dogs (48/53), 40/48 of which were spayed. Main breeds were Labrador (10/53), Australian Shepherd (4/53), and Miniature Schnauzer (3/53). A hooded vulva was noted in 33/48 of females. Transurethral cystoscopy showed anomalies in 45/53 of cases: mucosal edema (19/53), vestibulovaginal septal remnant (15/48), lymphoid follicles (8/53), short urethra (6/53), and ectopic ureter (5/53). Urine culture at the time of cystoscopy was positive in 13/49. Bladder wall edema and ulceration were the most common findings on histopathology (25/39).

**Conclusion and Clinical Importance:**

RUTI occurred more frequently in spayed female dogs. Transurethral cystoscopy is useful in the diagnosis and treatment of anomalies in dogs with RUTIs.

**Abbreviations**
EU: ectopic ureter
RUTI: recurrent urinary tract infections
TCC: transitional cell carcinoma
UTI: urinary tract infection
UVJS: ureterovesicular junction stenosis
VVSR: vestibulovaginal septal remnant

## 1 INTRODUCTION

Urinary tract infection (UTI) is common in dogs, especially in spayed females.^1^, ^2^ It is estimated that 5% to 27% of dogs will have at least 1 episode of UTI during their lifetime.^3^, ^4^ UTI results from a temporary or permanent breach in host defense mechanisms allowing adherence, multiplication, and persistence of microbial agents.^5^ These most common agents are fungi, viruses, mycoplasmas, parasites, and bacteria. Bacterial UTI is believed to result from bacterial organisms ascending via the urethra to the bladder from contamination via the gastrointestinal tract or skin surrounding the perineum. The presence of bacteria in urine does not equate to infection as 2.1% to 8.9% of female dogs have subclinical bacteriuria^6^, ^7^ that might prevent colonization by more pathogenic bacteria.^8^

Diagnosis of UTI relies on history, physical examination, complete urinalysis with sediment examination, and urine culture.^9^ Over‐treatment of dogs with subclinical bacteriuria is a frequent, inappropriate decision leading to over‐use of antibiotics.^10^

Recurrent UTI can be defined as either a relapse or reinfection.^11^ Relapse is defined by a recurrent infection with the same bacteria, most frequently associated with ineffective antimicrobial treatment. Reinfection is a recurrent infection with a different bacterial strain that usually indicates that host defense mechanisms are compromised. Identification of a breach in host defense mechanisms is crucial in order to prevent recurrence of UTI and thus development of bacterial resistance. Decreased frequency or incomplete voiding, damaged urothelium, and urethral sphincter incompetence can allow a greater number of bacteria to ascend and remain in the bladder. Congenital and acquired anatomic anomalies of the urogenital tract (urinary masses,^12^ uroliths,^13^ vestibulovaginal stenosis,^14^ vaginal strictures,^15^ ectopic ureters (EU),^16^ ureteroceles,^17^ hooded vulva,^15^ vestibulovaginal septal remnant (VVSR),^18^, ^19^ urethral septal membrane) can also predispose dogs to recurrent urinary tract infection (RUTI). Further investigations such as abdominal radiography, abdominal ultrasound, contrast imaging, and finally transurethral cystoscopy are indicated in the presence of RUTI.

Transurethral cystoscopy allows examination of the lower urinary tract, and identification of anatomic anomalies that can predispose to RUTI. Cystoscopy is an essential tool in interventional urology allowing correction of intramural ectopic ureters,^20^ fragmentation and removal of uroliths,^21^, ^22^ and laser ablation of polyps and vestibulovaginal septal remnants^19^ and urethral septums.

The objectives of this study were to describe the population of dogs presented to our hospital for RUTI that underwent transurethral cystoscopy and to compare them to the general population; as well as describe cystoscopic findings and minimally invasive correction of anatomic anomalies in dogs presented for RUTI.

## 2 MATERIALS AND METHODS

### 2.1 Definition of UTI

A UTI was defined as: presence of compatible clinical signs (ie, hematuria, pollakiuria, dysuria, stranguria) with concurrent evidence of bacterial cystitis based on at least 2 out of 3 of the following criteria: (1) pyuria (defined as >5 WBCs per high power field)^23^; (2) positive urine culture; (3) resolution of clinical signs with antibiotic treatment. Recurrence of UTI was defined as at least 2 episodes of UTIs within a 6‐month period or at least ≥3 in 1 year.

### 2.2 Case selection and data collection

Medical records of all dogs presented for RUTI that underwent transurethral cystoscopy at the Veterinary Teaching Hospital of the University of Montreal (CHUV) from January 1, 2011 to December 31, 2018 were retrospectively reviewed. Data collected from medical records included signalment, clinical presentation, physical examination findings, clinicopathologic/histopathologic, and cystoscopic findings. Age was approximated to the closest half year at the time of the first visit. Breeds were recorded and breeds with 3 or more (5%) individuals were compared to the reference population. Cases were excluded if they presented with subclinical bacteriuria or if they underwent a percutaneous cystolithotomy. The presence of a hooded vulva was defined on physical examination by a board‐certified internist. It was subjectively defined as the presence of a skin fold over the dorsal aspect of the vulva with less than approximately 50% of the vulva being visualized without any manipulation of the surrounding skin.

### 2.3 Cystoscopic procedure

Transurethral cystoscopy was routinely performed by 1 of 2 board‐certified internists (MD or CV). Dogs were anesthetized and placed in dorsal recumbency. Vulva, preputial, and perineal regions were clipped, aseptically prepared and a sterile drape was applied. A flexible cystoscope was used in male dogs (Storz© flexible 11278AUI/CE 0123 for cystoscopy before August 2018 then Storz© flexible Flex XC 11278/VSU #series SN25921, KARL STORZ Endoscopy Canada Ltd., Mississauga, Canada) and rigid cystoscopy was performed in female dogs (Wolf© rigid 8616.401/10.5 Fr used for female weighing less than 15 kg or Wolf© rigid 8642.403/14 Fr for heavier females >15 kg). The entire lower urogenital tract was systematically evaluated (prepuce, clitoral fossa, vestibule, urethra, bladder, ureteral papilla, vagina). When the urethra seemed subjectively short in regard to the dog size, the urethral length was measured from the proximal urethra (urethrovesicular junction) to the distal urethra (urethrovestibular junction). It was considered short when the length was inferior to 7 cm. We chose this length based on previous studies that measured mean normal urethral length of 8 cm.^18^, ^24^ Anatomic anomalies were noted and corrected during the same procedure (eg, ectopic ureters, vestibulovaginal septal remnant, urethral septum) using a Holmium:YAG or diode laser.

### 2.4 Bacterial culture and histopathologic findings

Urine samples for bacterial culture were obtained through the working channel of the cystoscope immediately upon entering the bladder. Bladder wall samples for histopathology were obtained at the end of the procedure using sterile cup biopsy forceps (Endoscopy Support Services 5 Fr x 65 cm, Brewster, NY) introduced through the working channel of the cystoscope. The area of sampling was chosen by the operator where the bladder wall was noted to be macroscopically abnormal while avoiding bladder trigone and the ureterovesical junction. First administration of peri‐operative antibiotics (Ampicillin 22 mg/kg q90min) was given at the time of introduction of the cystoscope.

### 2.5 Control population

In order to compare the study population to the overall population of dogs presented to the CHUV during the same study period, medical records of all dogs presented to the hospital were collected. Breed, sex, neuter status, and age at presentation were retrospectively obtained. The original visit was the only visit considered. Age was approximated to the closest half year at the time of the first visit. For dogs <3 months of age, 0 was assigned.

### 2.6 Statistical analyses

Descriptive statistics were used for signalment, clinical data, laboratory, and cystoscopic findings. All statistical analyses were performed with a commercial software program (SAS v.9.3. Cary, NC). An exact chi‐square test was used to compare prevalence of breed and sex distribution between the 2 study groups. An exact chi‐square test was also performed in order to evaluate a potential association between the administration of antibiotics before the procedure and the results of bladder wall culture. An unequal variance *t* test was used to compare the mean age between the 2 populations. A *P* value of less than .05 was considered statistically significant for all comparisons.

## 3 RESULTS

### 3.1 Study and reference population

A total of 53 dogs met the inclusion criteria and were included in the study population. This population included 48 (91%) female dogs, 40 of which (83%) were spayed females and 5 (10%) male dogs, 2 (40%) of which were neutered. The mean age at presentation of the study population was 3.64 ± 3.14 years with a median age of 3.0 years. A total of 35 different breeds were recorded, with Labrador Retrievers (n = 9), Miniature Schnauzers (n = 4), and Australian Shepherds (n = 4) being most common. A hooded vulva was present in 33/48 (69%) of female dogs.

During the study period, a total of 21 683 dogs were presented to the CHUV. The mean age at initial presentation was 5.0 ± 4.0 years with a median age of 4.0 years. This population was composed of 10 776 (49.6%) female dogs with 3540 (32.8%) intact and 7236 (67.2%) spayed. There were a total of 10 907 (50.4%) male dogs with 2860 (26.2%) intact and 8047 (73.8%) neutered.

The mean age was significantly lower in the study population than in the control population (*P* = .002). The reproductive status was also significantly different, with proportionately more intact females noted in the study population (*P* < .001). Labrador Retrievers (*P* = .05), Australian Shepherds (*P* = .01), and Miniature Schnauzers (*P* = .01) were overrepresented in the study population. RUTI were present in 0.25% (53/21683) of the general population during our study period.

### 3.2 Urinary tract infections clinical signs and diagnostic tests

The most common lower urinary tract signs noted in dogs in the study were pollakiuria (37/53, 70%), hematuria (23/53, 43%), dysuria/stranguria (13/53, 25%), urinary incontinence (30/53, 57%), and peri‐genital licking (23/53, 43%).

A total of 137 episodes of suspected UTI were reported in the medical file for the entire study population before presentation; each dog had at least 2 or 3 episodes depending on the time of recurrence (more or less than 6 months). Descriptions of each episode are described in Table 1.

**Table 1:** RUTI was defined by as at least 2 out of 3 criteria (1) compatible clinical signs; (2) pyuria; (3) positive bacterial culture

	Compatible clinical signs	Urinalysis	Bacterial culture
Number of episodes of UTI (total 137)	137/137	107/137	83/137
		Pyuria 104/107 (97.2%)	Positive: 73/83 (88%)
		Absence of pyuria 3/107 (2.7%)	Negative: 10/83 (12%)

*Note*: Results of the urinalysis and culture are summarized.

Sampling technique was recorded for every urine culture performed by the referring veterinarians. Seventy‐three positive urine cultures were performed. Fifty‐two (71%) were collected by cystocentesis, 11/73 (15%) were free flow urine samples and for 10 (14%) urine samples, the sampling technique was not recorded. *Escherichia coli* was most commonly cultured organism (49/73, 67%), followed by *Proteus mirabilis* (8/73, 11%), *Staphyloccocus* (8/73, 11%), and *Enterococcus* (3/73, 4%).

### 3.3 Cystoscopic findings

**Figure 1:**
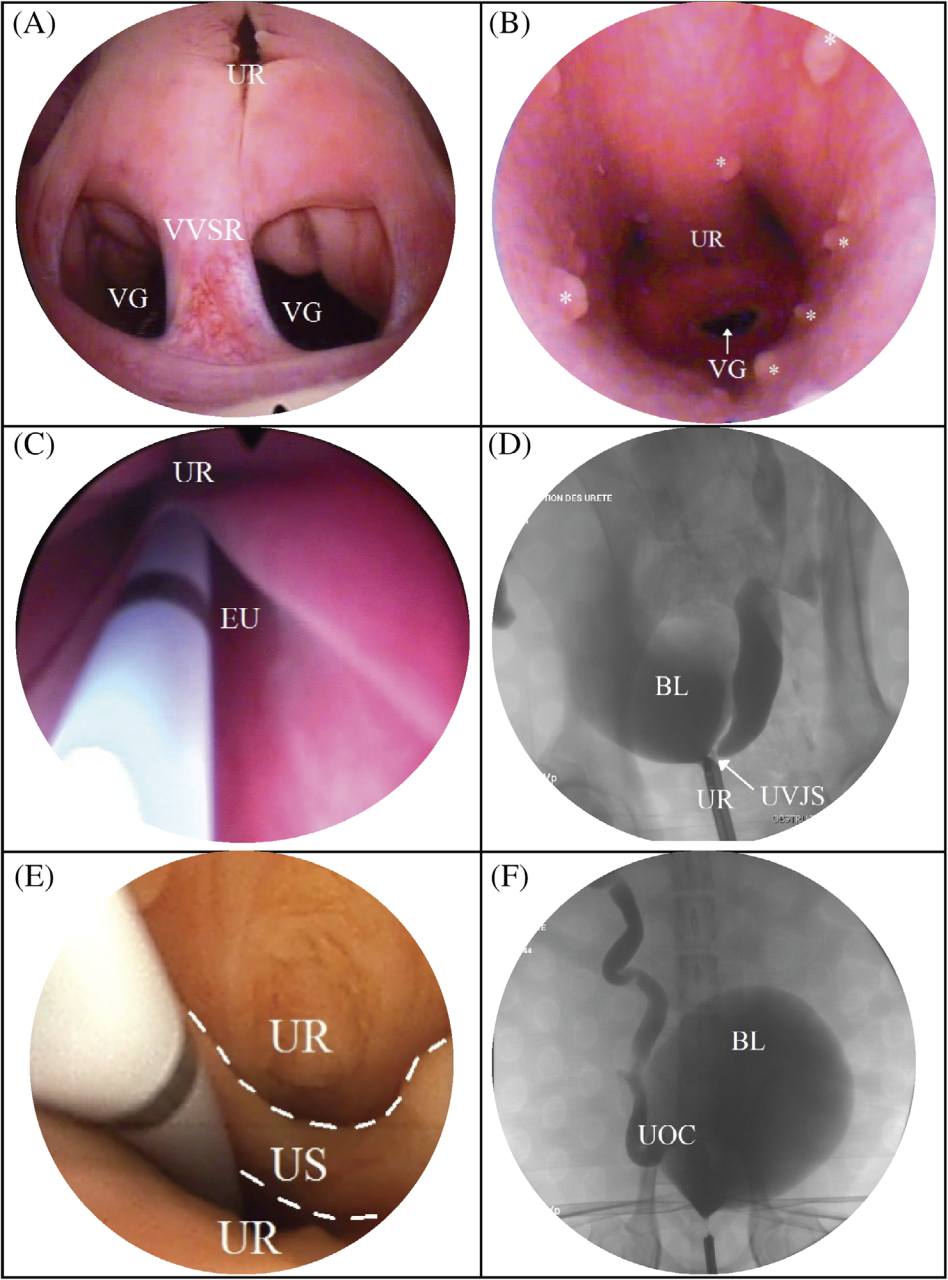
Cystoscopic and fluoroscopic images of anomalies seen in dogs presented for RUTI. A, Cystoscopic image of a vestibulovaginal septal remnant (VVSR), urethra (UR), and vagina (VG). B, Cystoscopic image of vaginal lymphoid follicles represented by asterisks. C, cystoscopic image of an ectopic ureter EU. D, Fluoroscopic image of a ureterovesical stenosis UVJS. E, Cystoscopic image of a urethral septum US, borders of the urethral septum are highlighted with white dashed lines. F, Fluoroscopic image of a ureterocele UOC with the bladder BL

Anomalies were seen in 45/53 (85%) of dogs (41/45 (91%) females; 4/45 (9%) males). Cystoscopic findings are described in Table 2. Ureterovesical junction stenosis was diagnosed in 3 dogs, 1 was bilateral, 1 was unilateral, and 1 was unilateral and associated with EU. In dogs with EU (n = 5), 4/5 were unilateral and 4/5 were noted to also have a vestibulovaginal septal remnant, all of them were presented with a history of urinary incontinence. Urethral septal membrane was only found in 2 male dogs. For the first male dog, a 2 cm membrane dividing the urethra in 2 different section along its longitudinal axis and was located just caudal to the prostatic urethra and cranial to the urethral flexure. For the second male dog, a septum dividing the urethra in 2 parts along its longitudinal axis was noted. The beginning of the septum was at the level of the mid‐penile urethra and finished at the level of the mid‐prostatic urethra. It was confirmed using fluoroscopy in this dog. Ablation of the septum in this second dog was performed on 2 different occasions because of iatrogenic inflammation induced by the laser ablation because of its extensive length. For both dogs, both ureteral papillas were noted to be correctly positioned in the urinary bladder trigone. Two cases of urethral stricture were reported but with variable severity. The first dog with urethral stricture was a neutered male. The stricture prevented the cystoscope from passing beyond the distal pelvic urethra. A voiding cystourethrogram was performed and revealed a 1.5 cm long stricture that required balloon dilatation in order to advance the cystoscope beyond the stricture. The second dog did not require urethral dilatation (see Figure 1).

**Table 2:** Dog data for the RUTI population divided by sex and neuter status

Dog data	Total females (n = 48)	Intact females (n = 8)	Spayed females (n = 40)	Total males (n = 5)	Intact males (n = 3)	Neutered males (n = 2)	Total
Mean age (years)	3.9	0.8	4.5	1.2	0.7	2	3.6
Clinical signs							53 (100%)
Pollakiuria	32	6	26	4	2	2	36 (70%)
Hematuria	23	2	21	…	…	…	23 (43%)
Dysuria/stranguria	15	1	14	…	…	…	15 (25%)
Urinary incontinence	25	5	20	5	3	2	30 (57%)
Peri‐genital licking	21	4	17	2	1	1	23 (43%)
Hooded vulva	33 (69%)	5	28	…	…	…	…
Anomalies	41 (85%)	6	35	4 (80%)	2	2	45 (85%)
Bladder mucosal edema	…	…	16	…	…	…	16
Urethral mucosal edema	…	…	2	…	1	…	3
VVSR	15	2	13	…	…	…	15
Lymphoid follicles	9	1	8	…	…	…	9
Short urethral length <7 cm	…	…	6	…	…	…	6
Ectopic ureter	5	1	4	…	…	…	5
Single bladder stone	…	…	3	…	…	…	3
Ureterovesicular junction stenosis	…	1	1	…	…	1	3
Urethral septum	…	…	…	…	2	…	2
Urethral stricture	…	…	1	…	…	1	2
TCC (urethral wall thickening)	…	…	1	…	…	…	1
Ureterocele	…	…	1	…	…	…	1
Urachal remnant	…	1	…	…	…	…	1
Bladder wall polyp (cranioventral)	…	…	1	…	…	…	1

Abbreviations: TCC, transitional cell carcinoma; VVSR, vestibulovaginal septal remnant.

In dogs with anomalies, 12/45 (27%) had nonspecific findings such as mucosal edema, prominent lymphoid follicles, or both of them.

Of the study population, 28/53 (53%) dogs underwent correction of their anomalies. Laser ablation of a vestibulovaginal septal remnant was done in 13/15 dogs. One vestibulovaginal septal remnant was removed with biopsy forceps and the other was associated with an urethrovaginal communication making laser ablation impossible. Cystoscopic laser ablation of UVJ stenosis was performed in 2/3 dogs, 1/3 underwent balloon dilatation. Cystoscopic laser ablation of a unilateral ectopic ureter (5/5), urethral septal membrane (2/2), orthotopic ureterocele (1/1), removal of a bladder polyp (1/1) were also performed. Laser lithotripsy was used to fragment a bladder stone in 2/3 dogs. The last dog had a small bladder stone that was flushed from the bladder. Stone fragments were removed by using a stone basket passed through the working channel of the cystoscope or by urohydropulsion. Injection of a urethral bulking agent was performed in 2 cases presented with urinary incontinence and without active UTI. Episioplasty was performed in 7/33 dogs; all were spayed females.

### 3.4 Histopathology

Biopsy samples were submitted for histopathology in 38 dogs (72%). Ulceration and mucosal edema were most commonly reported in 24/38 dogs (63%). No relevant anomalies were found in 11/38 dogs. Other findings included transitional cell carcinoma (1/38), hemorrhagic bladder polyp with hyperplastic lymphoid follicles in the urethra (1/38), and epithelial dysplasia and fibrosis (1/38).

### 3.5 Culture

Urine bacterial culture was performed in 49 (93%) (47 females, 2 males) dogs during the procedure. Twelve dogs had at least 1 positive culture. A total of 66 samples collected during the procedure were submitted for culture and sensitivity testing: 32/66 bladder mucosal biopsies, 29/66 urine samples, 2/66 bladder stones, 2/66 vaginal mucosal biopsies, and 1/66 urethra mucosal biopsy. A total of 17 samples cultured positive: 9/17 bladder wall biopsies, 7/17 urine samples, and 1/17 urethral mucosa. In 2 cases, the bladder wall culture was positive with a concurrent negative urine culture. In 1 case, *Enterococcus* was isolated in the bladder wall, in the other *Staphylococcus aureus*. One dog with an EU had a positive urine culture with an *E. coli* isolated, 3 had negative urine cultures and 1 had no culture submitted. Bacterial isolates obtained at the time of cystoscopy were *E. coli* 6/12 (50%), *Staphylococcus* 3/12 (25%), and *Enterococcus* 2/12 (17%).

## 4 DISCUSSION

RUTI were diagnosed in 0.25% of our general dog population which is similar to 0.3% previously decribed.^2^ Our prevalence might be slightly different because of our definition of UTI as compared to the referenced study. In our study, RUTI were defined as at least 3 successive positive urine cultures and the presence of clinical signs.^11^ In previous studies of RUTI, some dogs with subclinical bacteriuria were also included. Transient subclinical bacteriuria occurs in 8.9% of healthy female dogs.^7^

Female dogs were over‐represented in our study (91%), with a majority being spayed females (83%). This is also in agreement with previous reports.^1^, ^25^, ^26^, ^27^ Dogs presenting with simple UTI have a mean age of 7 to 8 years^1^, ^25^, ^28^ however age at presentation for RUTI varies widely^27^ which could reflect the broad spectrum of etiologies. In our study, the relatively young age of the population (3.78 years) can be explained by the detection of neoplasia with other imaging modalities (ultrasound/traumatic catheterization) at our facility. It has the advantage of being performed under sedation as opposed to undergoing cystoscopy under general anesthesia. In our population, Labrador Retrievers, Australian Shepherds, and Miniature Schnauzers were over‐represented. Similarly, in 3 previous studies, breeds varied widely but Labrador Retrievers are predisposed, representing more than 5% of cases.^2^, ^27^ Presence of a hooded vulva predisposes dogs to RUTI^18^, ^29^ that might explain its high prevalence in our study population (69%). Unfortunately, because of missing information in medical files, it was not possible to statistically compare age at spay between females with and without a hooded vulva.

One of the main nonspecific findings was prominent lymphoid follicles. Lymphoid follicles are not detected by radiographs nor CT scan.^18^ Though the clinical relevance of this finding remains unknown, cystoscopy is superior to other imaging modalities in their identification. Dogs with EU commonly have concurrent VVSR in 80% (4/5) which is similar to previous reports (83%).^19^ We observed a high prevalence of hooded vulva (69%); however, episioplasty was performed in only 21%. Episioplasty reduces UTIs in 84% to 100% of dogs,^29^, ^30^ likely by reducing bacterial population in the perivulvar region and therefore in the vulva and vestibule. We chose not to systematically perform episioplasty in dogs that were obese, had concurrent anomalies or were young intact females as these conditions might have predisposed to bacteria in the region. Alternatively, weight loss and allowing a female to come into heat is recommended.^31^ Episioplasty surgery was considered if UTIs continued to recur despite passing a heat cycle and undergoing correction of cystoscopically identified anomalies.

Our most frequent finding on histopathology was ulceration and mucosal edema (63%). We believe this finding could be related to chronic inflammation from the RUTI and filling the bladder with saline during the procedure that might alter the bladder epithelium. Further studies are required to differentiate these 2 hypotheses. If lesions are induced by filling the bladder with saline, biopsy sampling should be performed at the beginning of the procedure in order to minimize these changes. This can however limit examination because of bleeding and prolong the procedure time and thus anesthesia time. Biopsies were performed at the end of the procedure at our institution. Histopathology was nonspecific except for 1 case of transitional cell carcinoma.

The most common bacterial isolate was *E. coli*. *E. coli* is commonly cultured from urine of dogs with UTIs with a reported prevalence of 30% to 50% of urine cultures submitted.^13^, ^19^ The most prevalent bacteria after *E. coli* was *Staphylococcus* and *Enterococcus* as in 3 retrospective case studies.^2^, ^27^, ^32^ In our culture results, a single pathogen was present in 92% of dogs, with 2 pathogens isolated in only 1 urine sample. Sensitivity testing revealed that 7 out of the 12 positive urine cultures were resistant to at least 1 commonly administered oral antibiotic. In 2 dogs, urine culture was negative; however, bladder mucosal biopsy was positive which is in disagreement with previous reports.^13^, ^32^, ^33^ This discrepancy might be explained by the invasion of superficial bladder epithelial cells and submucosa by bacteria,^34^ even if the isolated bacteria in our cases were not *E. coli*, which has been previously described in host cells with type‐1 fimbriae‐mediated invasion. Even if this was present in a small number of dogs, we recommend urine culture and bladder mucosal biopsy culture in dogs undergoing cystoscopy for RUTI.

This study is limited by its retrospective nature and the small number of cases. The diagnosis and treatment of UTIs initially done by referring veterinarians was not standardized. It was not possible for us to differentiate relapse and reinfection as urine culture was not consistently repeated for every episode of UTI and clonality testing was not performed.^35^ Unfortunately, outcome and long‐term follow‐up were unable to be assessed given the retrospective nature of the study.

In conclusion, RUTI affect mainly spayed females (83%) with a mean age of 3.78 years. Labrador Retrievers, Australian Shepherds, and Miniature Schnauzers were over‐represented in our study. Transurethral cystoscopy is an effective, safe, and minimally invasive procedure to investigate RUTI. Anomalies were found in the majority of dogs (85%—45/53) with a correction performed at the time of cystoscopy in 53% (28/43).

## CONFLICT OF INTEREST DECLARATION

Authors declare no conflict of interest.

## OFF‐LABEL ANTIMICROBIAL DECLARATION

Authors declare no off‐label use of antimicrobials.

## INSTITUTIONAL ANIMAL CARE AND USE COMMITTEE (IACUC) OR OTHER APPROVAL DECLARATION

Authors declare no IACUC or other approval was needed.
